# Heightened expression of type I interferon signaling genes in CD4^+^ T cells from acutely HIV-1–infected women is associated with lower viral loads

**DOI:** 10.3389/fimmu.2024.1507530

**Published:** 2025-01-20

**Authors:** Elina El-Badry, Luxiao Chen, Khader Ghneim, Ziyi Li, Kelsie Brooks, Jake Rhodes, Rafick Sekaly, William Kilembe, Susan Allen, Hao Wu, Eric Hunter

**Affiliations:** ^1^ Emory Vaccine Center, Emory National Primate Research Center, Emory University, Atlanta, GA, United States; ^2^ Department of Biostatistics and Bioinformatics, Rollins School of Public Health, Emory University, Atlanta, GA, United States; ^3^ Department of Pathology and Laboratory Medicine, Emory University, Atlanta, GA, United States; ^4^ Center for Family Health in Zambia, Lusaka, Zambia

**Keywords:** HIV, sex-based differences, interferon, acute infection, transcriptomics

## Abstract

Sex differences play a role in the pathogenesis of a number of viral diseases. In HIV-1, several studies have reported that chronically infected women have significantly lower plasma viremia than men, although the exact mechanism by which this occurs has yet to be identified. We have performed bulk RNA-seq experiments comparing gene expression between CD4^+^ T cells from acutely HIV-1–infected men and women in Zambia, because we observe lower viral load (VL) despite higher CD4^+^ T-cell activation in these women during acute/early infection. In a univariate analysis, we have identified a number of differentially expressed genes in naïve, central memory, and effector memory CD4 T cells of women with consistent elevated expression of genes linked to type 1 interferon (IFN) signaling. Moreover, after controlling for differences in VL and CD4^+^ T-cell count, genes within the type I IFN signaling pathway were further shown to be more highly expressed in women, whereas those genes more highly expressed in men showed no such enrichment. A subset of the genes highly expressed in women was further identified, including several involved in type I IFN signaling in response to viral infections (*IRF7*, *DDX58*, *SAMHD1*, *OAS2*, and *TRIM14*), that both are more highly expressed in CD4^+^ T cells from women and negatively correlated with VL, suggesting that they play a role in the comparative control of VL observed in women.

## Introduction

Sex differences in pathogenesis of a number of viral diseases have been well-documented in the literature (1). In HIV-1, several studies have reported that chronically infected women have significantly lower plasma viremia than men (2–7). We have recently shown that this discrepancy in viral load (VL) begins during acute infection and remains consistent throughout the duration of infection (8). However, the exact mechanism by which women maintain consistently lower VL than men, despite more activated CD4^+^ T cells early in infection, has yet to be elucidated. Here, we describe gene expression analysis of CD4^+^ T cells from acutely HIV-1–infected men and women to identify genes that may contribute to this phenomenon.

During HIV-1 infection, type I interferons (IFNs) engage in a number of antiviral activities including degradation of RNA, arrest of cell cycle progression, promotion of antigen presentation, and induction of apoptosis in infected cells while simultaneously contributing to the induction of chronic immune activation (9, 10). This is evident in the rhesus macaque model, where IFNα administration was shown to initially upregulate expression of IFN-stimulated genes (ISGs) and to prevent systemic SIV infection. However, continued IFNα treatment also resulted in accelerated CD4^+^ T-cell depletion and increased viremia (11). In agreement with the results obtained from the primate models of SIV infection, several studies have reported a strong activation of different components associated with the type I IFN response (including Interferon regulatory factors (IRFs), interferon-stimulated genes (ISGs) and viral DNA sensors) during chronic HIV-1 infection (12–16). High levels of ISGs such as Interferon-gamma inducible protein 10 (IP-10) are also associated with more rapid CD4^+^ T-cell depletion (17, 18).

Previous gene expression analysis demonstrated clearly that expression of ISGs increases in HIV-infected individuals in various cell types (13, 15, 19, 20). However, a study of chronically HIV-1–infected individuals found that several ISGs were expressed at significantly higher levels by CD4^+^ T cells [as well as CD8^+^ T cells and plasmacytoid dendritic cells (pDCs)] from women than men but only after correcting for differences in VL (21). It has additionally been demonstrated that pDCs from women produce significantly higher amounts of IFNα than those from men after stimulation with HIV-1 antigens, implying a more activated phenotype given similar antigenic stimulation (22). The basis for this appears to be escape from X-chromosome inactivation in pDCs of the gene for the Toll-like receptor, TLR7, resulting in bi-allelic expression of the gene and higher levels of expression of IFNα and IFNβ (23). It is therefore conceivable that both female sex and antigenic stimulation with HIV-1 contribute to expression of ISGs that play a role in the immune response to HIV-1 infection.

Much of the work describing sex differences during viral infection focuses on pDCs, the primary producers of IFNα. Comparatively, little is known about sex differences in gene expression between CD4^+^ T cells, the primary target cell of HIV-1. Additionally, previous studies of sex differences in HIV-1 pathogenesis have focused on the chronic phase of infection or in antiretroviral therapy (ART)–treated individuals. Here, we describe experiments comparing gene expression between CD4^+^ T cells from acutely HIV-1–infected men and women in Zambia who were ART naïve at the time of sample collection, allowing for comparison of natural infection in the absence of treatment. We identify a subset of genes, including several involved in type I IFN signaling, that are both more highly expressed in CD4^+^ T cells from women and negatively associated with VL, suggesting that they play a role in the comparative control of VL observed in women.

## Results

### Type I IFN*–related genes are differentially expressed between CD4^+^ T cells of acutely HIV-1–*infected men and women

We performed bulk RNA sequencing (RNA-seq) on Fluorescence-Activated Cell Sorted (FACS) CD4^+^ T cells from cryopreserved peripheral blood mononuclear cells (PBMC) of four men and four women acutely infected with HIV-1 [approximately 3 months post–estimated date of infection (EDI); median, 86.5 days post-EDI]. All subjects were enrolled in the International AIDS Vaccine Initiative (IAVI) Protocol C in Lusaka, Zambia, and were ART naïve at the time of sampling, in compliance with standard of care in Zambia at the time of sample collection (2006–2009). Additional subject demographic information is listed in **Table 1**.

**Table 1:** Subject information.

Subject ID	Sex	Age	Days post-EDI^a^	Log_10_VL^b^	Log_10_SPVL^c^	CD4 count (cells/mm^3^)
235095	F	22	49	5.74	5.44	475
305123	M	29	82	5.57	5.62	284
235072	M	26	87	5.16	5.47	335
235216	M	29	86	4.52	4.43	515
235092	M	27	112	4.20	4.30	527
235074	F	25	89	4.07	4.60	525
235086	F	23	84	3.21	3.71	738
235036	F	35	138	3.00	5.00	545

a EDI, Estimated date of infection.

b VL, viral load.

c SPVL, set-point viral load.

Demographic and sample information from eight subjects included in RNA-seq experiments.

Live CD4^+^ T cells were sorted into effector memory (T_EM_; CCR7^−^CD45RA^−^), central memory (T_CM_; CCR7^+^CD45RA^−^), and naïve populations (T_naive_; CD27^+^CD45RA^+^). An average of 15,000 cells was sorted for RNA extraction and cDNA synthesis, and sequencing at a depth of 17–20 million reads per sample was performed. Dimension reduction via multi-dimensional scaling revealed that the largest transcriptomic difference was driven by sorted cell type (**Supplementary Figure S1A**). Differences between male and female participants were observed inside each subset. We assessed the quality of the samples and tested for the specific expression of X and Y chromosome genes before moving to the downstream analysis and observed elevated levels of *XIST* and *DDX3Y* in female and male participants, respectively (**Supplementary Figure S1B**).

**Figure 1:**
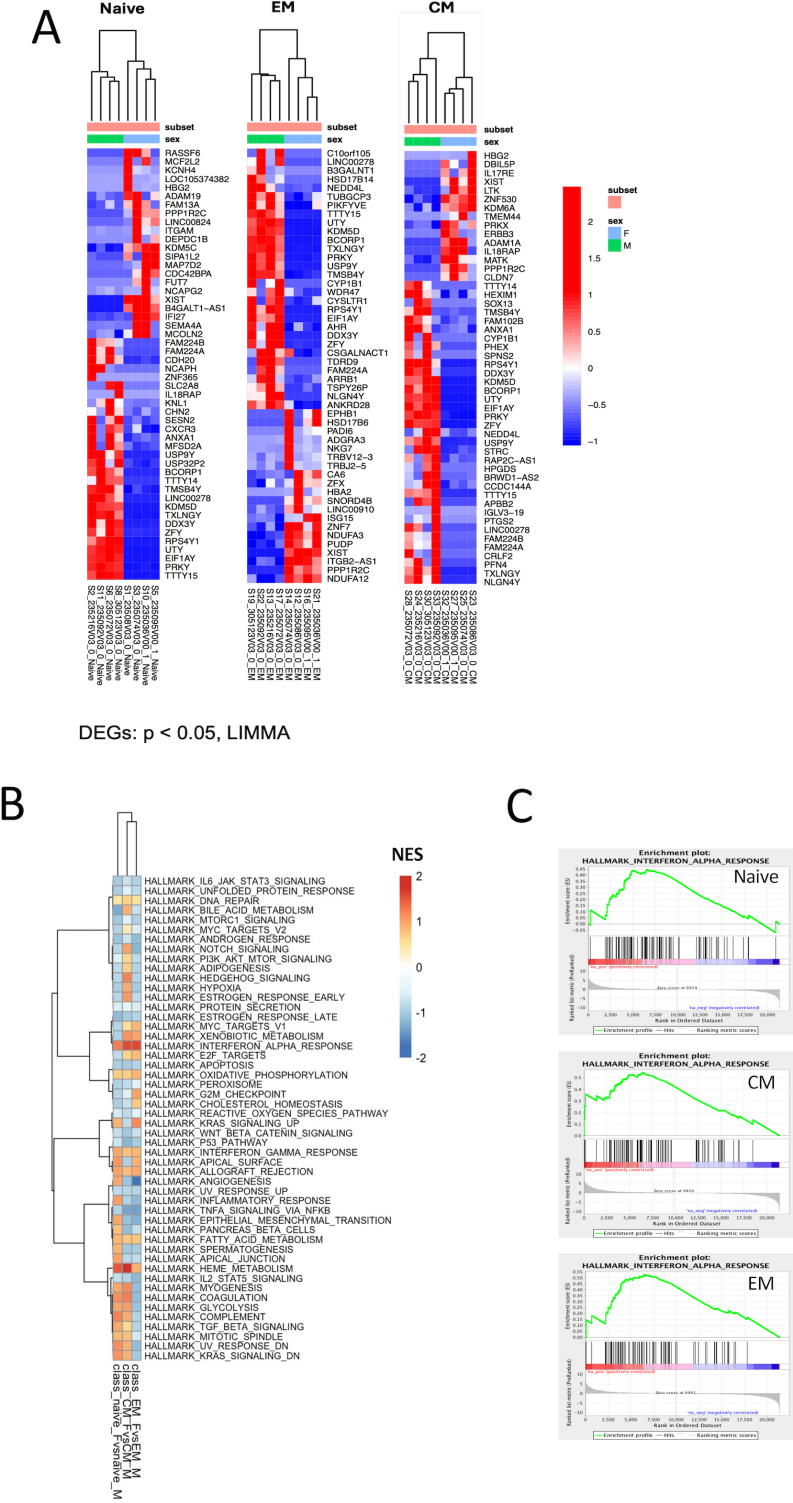
Differential expression of genes between CD4^+^ T cells of acutely HIV-1–infected men and women. **(A)** Heatmaps representing the top 50 differentially expressed genes (DEGs) between men and women in naïve, EM, and CM sorted cell subsets. Rows represent genes (p < 0.05), and columns represent samples. Gene expression is represented as a gene-wise standardized expression (Z-score). Red and blue correspond to up- and downregulated genes respectively. **(B)** Gene set enrichment analysis (GSEA) was used to identify pathways that demarcate female and male participants in naïve, EM, and CM sorted subsets. Induced and repressed pathways from the Hallmark database are plotted along the y-axis. NES score with red and blue squares reflects positive and negative enrichment, respectively. **(C)** Gene enrichment plots of the interferon-α pathway are shown for each of the T-cell subsets. A list of DE genes for each CD4 T cell subset is shown in **Supplementary Tables S2** (naïve), **S3** (EM), and **S4** (CM). The results of GSEA for each CD4 T cell subset are shown in **Supplementary Table S5** (naïve), **S6** (EM) and **S7** (CM).

**Figure 2:**
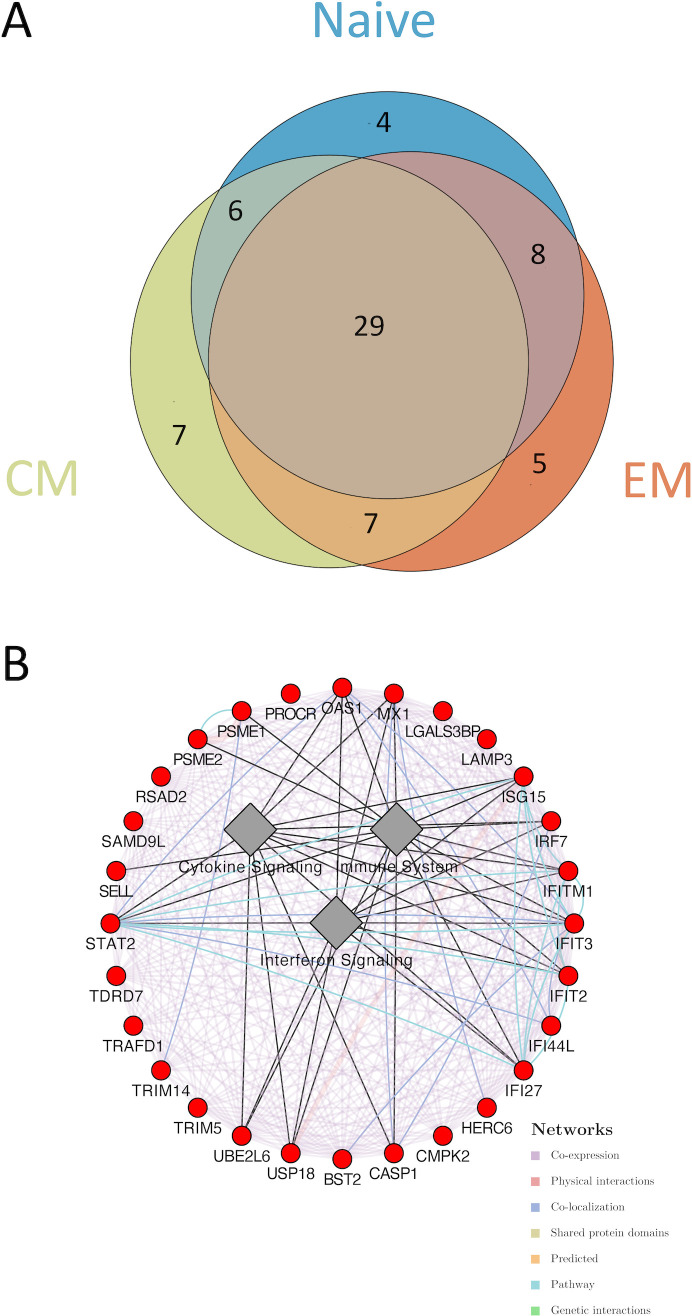
Differential gene enrichment analysis between CD4 subsets in acutely HIV-1–infected women and men. **(A)** A Venn diagram illustrating the overlap of leading-edge genes of the interferon signaling pathways upregulated in female participants between naïve, EM, and CM subsets. **(B)** A co-expression network was plotted to highlight the common genes. Cytoscape was used to plot the co-expression network highlighting the top leading-edge genes significantly enriched. GeneMANIA algorithm was used to infer network connections and co-expression. Circular nodes represent genes and edges reflect the association between these features. Nodes are colored in red to indicate upregulation in female participants. Biological annotation (diamond nodes) reflecting function of sets of genes is represented.

Further unsupervised analysis of the individual sorted CD4 T-cell subsets revealed different transcriptomic signatures segregating male and female participants in naïve, EM, and CM cell subsets (**Figure 1A**). Global pathway analysis comparing female and male participants revealed a strong induction of IFN signaling that was upregulated in women in all subsets. In contrast, inflammatory pathways such as TNFα signaling in women were downregulated (**Figures 1B, C**). These differences were highly significant for both the CD4_CM_ and CD4_EM_ subsets (**Supplementary Table S1**). Further investigation of IFN induction observed in women revealed the enrichment of 29 genes that were common across all three T-cell subsets (**Figures 2A, B**). This included an enrichment of several innate anti-viral host restriction factors (*IRF7*, *ISG15*, *MX1*, *OAS1*, and *IFITs*) downstream of IFN signaling in female participants (**Figure 2B**). IRF7 is a master regulator of type I IFN production downstream of viral sensors (24). *ISG15* encodes an E3 ubiquitin ligase with anti-HIV activity, and *USP18* encodes an ISG15-specific protease, serving as a counterweight to ISG15 function (25). These results highlight that women maintain a better innate anti-viral response when compared to men.

Data generated by combining read counts from these three sorted populations are referred to as total CD4^+^ T cells. Analysis of this combined dataset found that 168 genes were more highly expressed in women than men (**Supplementary Figure S2A**). As would be predicted from the analysis of individual subsets, genes in the type I IFN signaling pathway were expressed at significantly higher levels in women than those in men. These included *IRF7*, *USP18*, *ISG15*, *IFI27*, and *IFI6* (**Supplementary Figure S2B**). In men, 308 genes were expressed by total CD4^+^ T cells to a greater extent than in women. In this gene set, we found modest enrichment for genes associated with both positive and negative regulation of T-cell activation as well as positive and negative regulation of apoptotic processes.

### Immune system signaling pathways are significantly enriched in genes more highly expressed by CD4^+^ T cells from women after controlling for VL and CD4^+^ cell count

Previous studies have demonstrated that healthy as well as acutely HIV-1–infected women have significantly higher numbers of CD4^+^ T cells than men (8, 26). Women have additionally been demonstrated to have consistently lower VL than men throughout HIV-1 infection (2–8). Therefore, in order to understand how sex affects CD4^+^ T-cell gene expression in the absence of these differences in viral stimulation and target cell number, we performed a multivariate regression analysis examining differential expression of genes while controlling for CD4^+^ T-cell count and VL of each individual at the time of sampling.

**Figure 3:**
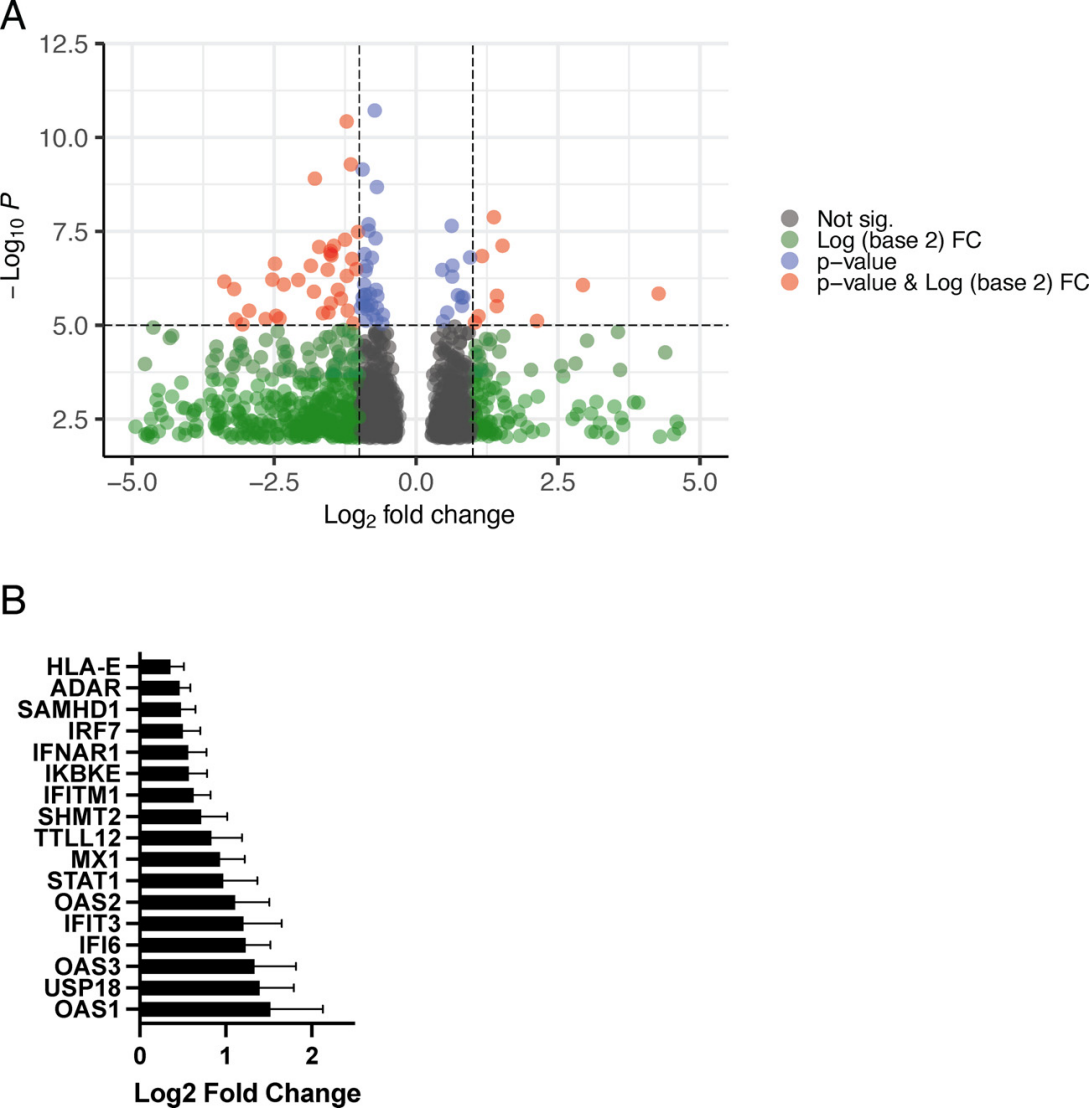
Differential expression of genes between men and women when controlling for SPVL and CD4^+^ T-cell count. **(A)** Differential expression of genes in women compared to those in men (FDR < 0.20, p-value < 0.05). Further cutoff values are plotted: p-value cutoff value set to 10e-6 (−log_10_P = 5) (blue), log_2_ fold change set to |1| (green), both p-value cutoff and log_2_ fold (red), and not significant (gray). **(B)** Differential expression of type I IFN–responsive genes in women (GO:0060337, p_adj_ = 7.95 × 10^−6^).

In this analysis, we found that 710 genes were more highly expressed by total CD4 T cells in women than men (**Figure 3A**). Functional analysis of this gene set found significant enrichment for genes involved in the response to type I IFN including a number of genes that encode proteins with direct antiviral function such as *IFITM1*, *SAMHD1*, *MX1*, and *OAS1-3* (**Figure 3B**). Importantly, the proteins encoded by *IFITM1* and *SAMHD1* have direct anti-HIV function, interfering with virus entry and replication, respectively (27–29). Other genes that meet this criteria code for proteins that are directly involved with type I IFN signal transduction include *IFNAR1*, *IRF7*, and *STAT1*. A total of 1,182 genes were more highly expressed by CD4^+^ T cells from men than those from women; however, in this gene set, we found no enrichment for pathways involved in immune system function; rather, those involved in metabolism, RNA biosynthesis, and processing were identified.

### Expression of multiple genes is significantly associated with VL

**Figure 4:**
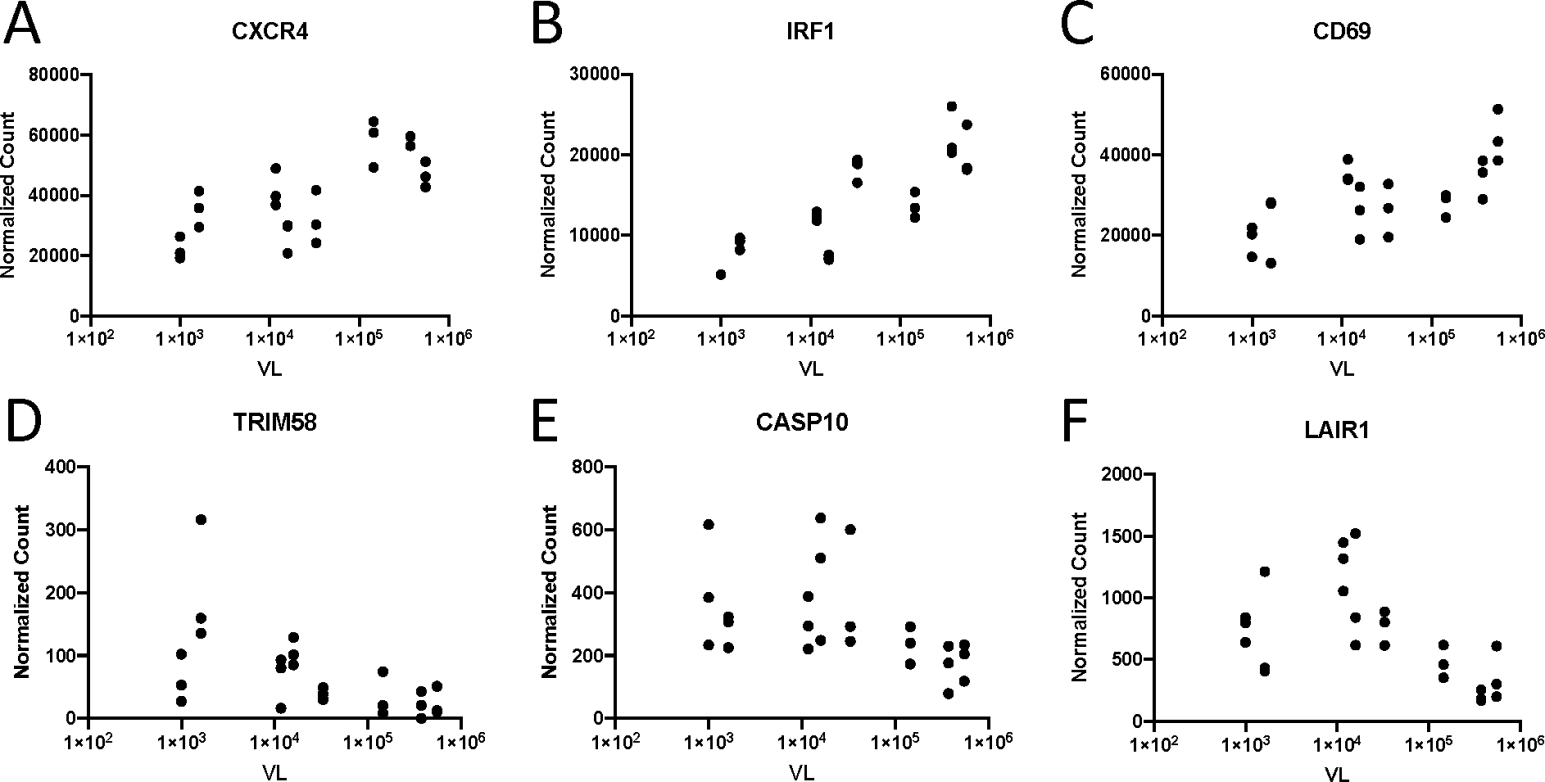
Expression of selected genes correlated with VL. Normalized expression of **(A)** CXCR4 (p_adj_ = 0.036), **(B)** IRF1 (p_adj_ = 0.004), **(C)** CD69 (p_adj_ = 0.024), **(D)** TRIM58 (p_adj_ = 0.034), **(E)** CASP10 (p_adj_ = 0.016), and **(F)** LAIR1 (p_adj_ = 0.002) versus subject viral load. Data points with identical VL represent normalized gene expression CD4^+^ T_naive_, T_EM_, and T_CM_ subsets from a single subject.

In order to understand how gene expression contributes to differential VL observed in men and women, we first performed a univariate analysis of VL versus gene expression. Using a generalized linear model framework, we analyzed differential expression of genes per unit change in VL. Using this model, we found differential expression of 2,097, whose expression was significantly correlated with VL. A total of 909 of these were positively correlated with VL (positive log_2_ fold change per unit increase in VL). Selected examples of these correlations are shown in **Figure 4**. *CXCR4*, which encodes one of the two primary co-receptors for HIV-1 and is endowed with potent chemotactic activity for lymphocytes, was positively correlated with VL (**Figure 4A**). Additionally, a number of genes in the type I IFN signaling pathway are also positively correlated with VL including *IRF1* (**Figure 4B**). This agrees with numerous studies, as IFN expression is stimulated by viral sensing (13, 15, 19, 20). Furthermore, expression of a number of genes encoding T-cell activation molecules is positively associated with VL including *CD28* and *CD69*, consistent with the fact that HIV-1 induces significant T-cell activation (**Figure 4C**) (30).

We additionally found that expression of 1,188 genes was negatively associated with VL (negative log_2_ fold change per unit increase in VL). Several members of the TRIM family of E3 ubiquitin ligases including *TRIM2*, *TRIM14*, *TRIM27*, *TRIM46*, *TRIM58*, and *TRIM62* (**Figure 4D**) were all negatively associated with VL. Furthermore, we found that genes associated with cell death including *CASP10* (**Figure 4E**) and *CASP1* and expression of inhibitory receptors including *LAIR1* (**Figure 4F**), *CISH*, and *SOCS2* were also negatively associated with VL.

### Fifty-one genes more highly expressed in women are also associated with lower viral load

**Figure 5:**
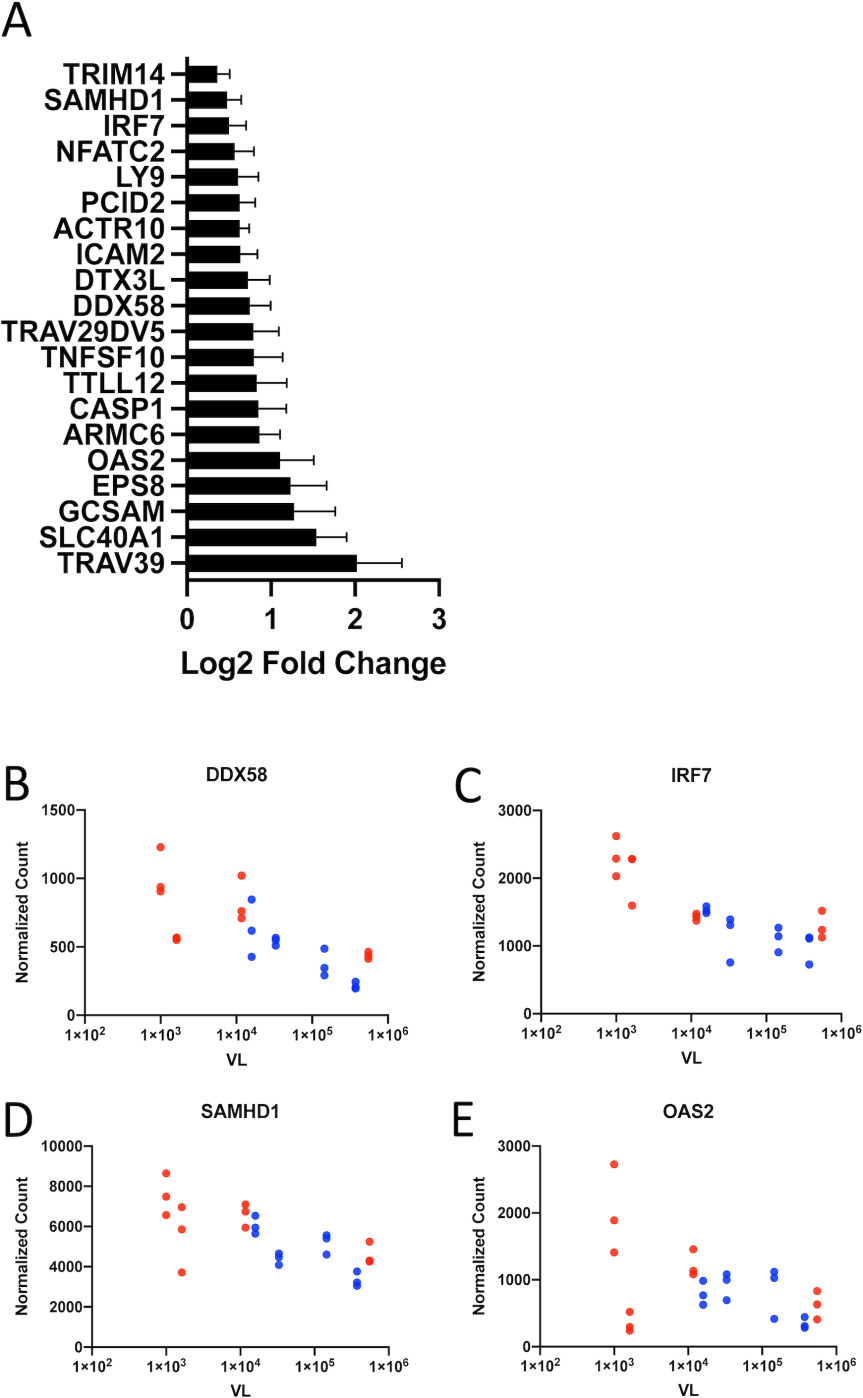
Genes more highly expressed in women are also associated with lower viral load. **(A)** Genes that are both more highly expressed in CD4^+^ T cells from women and inversely correlated with viral load and fall under the GO term GO:0002376, Immune System Process. Representative graphs of these genes involved in type I interferon signaling: **(B)** DDX58, **(C)** IRF7, **(D)** SAMHD1, and **(E)** OAS2. Data from female subjects are shown in red and data from male subjects in blue. Data points with identical VL represent normalized gene expression CD4^+^ T_naive_, T_EM_, and T_CM_ subsets from a single subject.

In order to understand how genes expressed, to a greater extent, in women can contribute to lower VLs, we determined which genes were expressed more highly in women and were also associated with lower VL. Fifty-one genes met these criteria. The full list of differentially expressed genes that are both more highly expressed in women and negatively associated with VL can be found in **Supplementary Table S8**. This gene set contains significant enrichment for immune system processes (GO:0002376, p_adj_ = 0.032) (**Figure 5A**). These include cell death pathways (*CASP1*, *TNFSF10*, and *BCL2L1*) as well as two signaling lymphocytic activation maker (SLAM) family members: *SLAMF6* and *LY9*. It also includes two genes that encode antiviral E3 ubiquitin ligases, *DTX3L* and *TRIM14*; the latter, in particular, has been reported to be an IFN-stimulated HIV-1 restriction factor (31). Several other genes in this set code for genes that are involved in various stages of type I IFN signaling. *DDX58* encodes the viral RNA sensor RIG-I, which triggers downstream IFN signaling cascades (**Figure 5B**) (32). *IRF7*, as described earlier, is a master regulator of type I IFN production critical for the antiviral response (**Figure 5C**) (24), whereas *SAMHD1* and *OAS2* are ISGs with direct antiviral function (**Figures 5D, E**).

## Discussion

We have demonstrated here that a significant number of genes involved in important immune system functions are differentially expressed in the CD4^+^ T cells of women compared to those of men during acute/early HIV-1 infection. Many of these differences in expression can be observed without controlling for the known differences in HIV-1 VL and CD4^+^ T cells that exist between men and women. However, by controlling for these factors, we can identify a large number of genes and specific functional pathways that may directly contribute to control of VL commonly observed in HIV-1–infected women.

Without controlling for VL and CD4^+^ cell count, we have identified 476 genes that are differentially expressed between CD4^+^ T cells of acutely HIV-1–infected men and women. Several genes involved in the type I IFN signaling pathway were expressed at significantly higher levels in women than those in men, both in total and individual CD4 T-cell subsets (EM, CM, and naïve). This is consistent with previous reports that ISGs were more highly expressed from CD4^+^ T cells (in addition to CD8^+^ T cells and pDCs) in chronically infected women than men (21). Specifically, we confirm that *ISG15*, a known HIV-1 restriction factor, is more highly expressed in CD4^+^ T cells in women. Importantly, we show here that these differences in expression are present from early in infection and may therefore set the stage for later differences in pathogenesis. We also identify heightened expression of additional IFN signaling genes such as *IRF7*, *USP18*, *IFI27*, *IFI6*, *MX1*, *OAS1*, and numerous *IFITs*, many of which are known innate anti-viral host restriction factors. This demonstrates heightened expression in women of genes throughout the type I IFN signaling pathway including a master regulator of type I IFN production (*IRF7*) critical for the antiviral response (24). Interestingly, whereas, in women, genes associated with an IFNα response were consistently elevated in all CD4 T-cell subsets, those associated with TNFα signaling were consistently reduced compared to those in men. This is consistent with a recent study of women on anti-retroviral treatment that showed inverse correlations between IFNα and TNFα responses of pDCs to Toll-like receptor 7/8 stimulation (33).

When controlling for differences in VL and CD4^+^ cell count between men and women, the differences in gene expression are even more apparent. We find significant enrichment for a number of immune signaling pathways among genes more highly expressed in women, particularly those involved in the response to and production of type I IFN. In this multivariate regression model, we identified 710 genes were more highly expressed by CD4^+^ T cells from women than those from men. In this gene set, we found significant enrichment for genes involved in the response to type I IFN, including a number of genes that code for proteins with direct antiviral function: *USP18*, *IFITM1*, *SAMHD1*, *MX1*, and *OAS1-3*. Others are directly involved with IFN signal transduction including *IFNAR1*, *IRF7*, and *STAT1*. Whereas an even greater number of genes were more highly expressed by CD4^+^ T cells from men, we found no enrichment for pathways involved in immune system function, highlighting that this heightened type I IFN response to acute infection is specific to women.

Importantly, we have also identified a large number of genes whose expression was directly correlated with VL regardless of patient sex. Many of these are validated by previous work. For example, expression of ISGs is known to increase in HIV-infected individuals in various cell types (13, 15, 19, 20). As would be expected, we find that some ISGs, including *IRF1* and *IRF8*, are positively associated with VL. Furthermore, a number of T-cell activation molecules are positively associated with VL including *CD28* and *CD69*.

However, we also identified a number of ISGs whose expression negatively correlates with VL, including *IRF7* and *IRF3*. Type I IFNs engage in a number of antiviral activities; however, they simultaneously contribute to the induction of chronic immune activation (10). This is evident in the rhesus macaque model, where IFNα administration was shown to initially upregulate expression of ISGs and to prevent systemic SIV infection, but continued IFNα treatment also resulted in accelerated CD4^+^ T-cell depletion and increased viremia (11). It is possible that the ISGs negatively correlated with VL are those that impart sufficient restriction on viral replication, although additional experimentation *in vitro* will be required to discern the causality of these relationships.

Nevertheless, many of the identified genes have no known relationship to HIV-1 pathogenesis. For example, we identified a number of TRIM E3 ubiquitin ligases (*TRIM2*, *TRIM27*, *TRIM46*, *TRIM58*, and *TRIM62*) whose expression negatively correlated with VL that have not been previously implicated in restricting HIV-1 replication. Further analyses and experimentation are likely to reveal additional potential regulators of HIV-1 pathogenesis.

Finally, we demonstrated that a number of genes that are more highly expressed by CD4^+^ T cells in women than those in men during acute infection were also significantly correlated with lower VL. This gene set is significantly enriched in genes involved in the response to type I IFN including *IRF7*, *DDX58*, *OAS2*, *TRIM14*, and the HIV-1 restriction factor *SAMHD1*. Although CD4^+^ T cells are not the primary producers of type I IFN, it is possible that the effects of differential type I IFN production by pDCs between the sexes result in the disparate gene expression that we observed here (34).

*CASP1*, which encodes the pro-inflammatory Caspase-1, was additionally more highly expressed by women and inversely correlated with VL. Our previous data demonstrate that women, despite starting with higher numbers, lose CD4^+^ cells during HIV-1 infection at over twice the rate of men (8). Identification of *CASP1*, which mediates cell death through pyroptosis, in this group of genes suggests that this death mechanism may play a role in the increased rate of CD4^+^ cell loss in women. Although the exact mechanisms of CD4^+^ T-cell loss during HIV-1 infection have not been fully elucidated, recent reports suggest that the Caspase-1–mediated pyroptosis provides the main mechanism (35). It remains to be seen whether other markers of pyroptosis are also increased in CD4^+^ T cells from HIV-1–infected women compared to those from men, particularly at the protein level.

It should be noted that the small sample size of this exploratory study is a limitation that was imposed by sample availability at the time the study that was initiated. Moreover, one of the female subjects, PCID235095, represented an outlier in that she had the highest VL at the time of sample collection of all of the subjects, with lower expression of genes inversely associated with VL. This may have reduced the overall differences in gene expression between the men and women in the study. Nevertheless, taken together, these data help to elucidate important differences in gene expression during the acute phase of HIV-1 infection and identify several antiviral and pro-inflammatory genes that may contribute to the consistent control of viral replication observed in HIV-1–infected women.

## Materials and methods

### Study subjects

All participants in the Zambia Emory HIV Research Project discordant couple cohort in Zambia and IAVI Protocol C were enrolled in human subject protocols approved by both the University of Zambia Research Ethics Committee and the Emory University Institutional Review Board. Before enrollment, individuals received counseling and signed a written informed consent form agreeing to participate. All subjects selected for this study were ART naïve at the time of sample collection in accordance with standard of care and ART availability at the time (2006–2009). None of the female participants included in assays described were pregnant at the time of sample collection. The algorithm used to determine EDI has been previously described (36). Set-point VL (SPVL) was calculated as the mean of log_10_ VL values between 30 and 365 days post-EDI.

### Cell sorting and RNA-seq

Cells were sorted on a FACSAria III (BD, Franklin Lakes, NJ). Markers used for sorting are shown in **Supplementary Table S9**. Cells were sorted into CD4^+^ T_naive_ (CD27^+^CD45RA^+^), T_EM_ (CCR7^−^CD45RA^+^), and T_CM_ (CCR7^+^CD45RA^−^) subsets. Total CD4^+^ T cells refer to data combining normalized gene expression from these subsets. An average of 15,000 cells were sorted at 4°C into ribonuclease (RNase-free) Eppendorf tubes containing Roswell Park Memorial Institute Medium (RPMI). Cells were then centrifuged at 600 x g for 10 min, and supernatant was removed. Buffer RLT (350 µL; Qiagen, Hilden, Germany) plus 1% β-mercaptoethanol (BME) was added, and cells were vortexed for 1 min. Lysed cells were then stored at −80°C and submitted to the Emory NHP Genomics Core for RNA extraction.

Cells were lysed in 350 µL of Buffer RLT and then extracted using the RNeasy Micro kit (Qiagen) with on-column DNase digestion. RNA quality was assessed using a Bioanalyzer 2100 (Agilent), and then 500 picograms of total RNA was used as input for cDNA synthesis using the Clontech SMART-Seq v4 Ultra Low Input RNA kit (Takara Bio) according to the manufacturer’s instructions. Amplified cDNA was fragmented and appended with dual-indexed barcodes using the Nextera XT DNA Library Preparation kit (Illumina). Libraries were validated by capillary electrophoresis on a TapeStation 4200 (Agilent), pooled at equimolar concentrations, and sequenced with SE100 reads on an Illumina NovaSeq 6000, yielding ~25 million reads per sample on average.

### Data analysis

#### CD4 subset analysis

Total RNA-seq was completed. Trimming (Trimmomatic v0.33), Alignment (STAR v2.4.2a), and Counting (HTSeq v0.6.1) were performed, and all samples had sufficient counted reads after aligning to the human genome, which provided ample coverage to perform downstream differential expression analysis. Downstream analysis of the RNA-seq data was done using the R statistical language (37) and the Bioconductor suite (38). The LIMMA package (39) was used to fit linear models to and to perform a (moderated) Student’s *t-*test to assess the association of gene-expression between female and male participants in a specified cell subset. For data mining and functional analyses, genes that satisfied a p-value of <0.05 were selected. When indicated, the proportions of false positives were controlled using the Benjamini and Hochberg method.

##### Pathway analysis

Gene set enrichment analysis (GSEA) (40) using the Hallmark (MSigDB) was performed to identify pathways distinguishing women and men in naïve, CM, and EM sorted subsets. GSEA is a statistical method to determine whether members of a particular gene set preferentially occur toward the top or bottom of a ranked-ordered gene list where genes are ranked by the strength of their association with the outcome of interest. More specifically, GSEA calculates a normalized enrichment score (NES) that reflects the degree to which a set of genes is overrepresented among genes differentially expressed. The significance of an observed NES is obtained by permutation testing: resorting the gene list to determine how often an observed NES occurs by chance. Leading edge analysis is performed to examine the particular genes of a gene set contributing the most to the enrichment.

##### Network *mapping/*visualization

GeneMANIA Networks (41) (Genemania.org) were plotted to represent co-expression of genes. Overlap between the genes included in the networks, and Gene Ontology (GO) biological process was assessed using a Fisher exact test. GeneMANIA is a flexible, user-friendly web interface for generating hypotheses about gene function, analyzing gene lists and prioritizing genes for functional assays. Given a query list, GeneMANIA extends the list with functionally similar genes that it identifies using available genomics and proteomics data. GeneMANIA also reports weights that indicate the predictive value of each selected data set for the query. The Cytoscape (cytoscape.org) plugin was used to plot the networks.

#### Combined total CD4 analysis

Sequencing reads were aligned to a reference genome GRCh38 using STAR (42). Gene level read counts were obtained using HTSeq (43) Differential expression analysis was then performed using DESeq2, including internal normalization to correct for library size and shrinkage estimation for dispersions and fold changes (44). DE genes between men and women were separately detected either by comparing the two groups directly (univariate analysis) or by controlling effects of VL and CD4^+^ cell count (setting them as covariates in experimental design of DESeq2) (multivariate analysis). Genes with adjusted false discovery rates (FDRs) less than 0.20 and p-value less than 0.05 were deemed differentially expressed (DE). By setting VL as the only covariate in experimental design of DESeq2, the selected DE genes are thought as statistical significantly correlated with VL (positive log_2_ fold change represents a positive correlation, whereas negative log_2_ fold change represents a negative correlation). Volcano plots were generated using EnhancedVolcano (45) in R Studio Version 1.2 (Boston, MA). GSEA was performed using DAVID Functional Annotation Tool version 6.8 (46, 47). Bar plot of enriched terms was generated using GOplot (48).

## Data Availability

The data presented in this study have been deposited in the GEO repository, accession number GSE285839.
