# Supplementary material for: Heightened expression of type I interferon signaling genes in CD4+ T cells from acutely HIV-1–infected women is associated with lower viral loads
Source: Front Immunol. 2025 Jan 20;15:1507530. doi: 10.3389/fimmu.2024.1507530 (PMC11788160; doi:10.3389/fimmu.2024.1507530)
Supplement: Supplementary file 1 [file Image1.pdf]

Supplementary Material

A

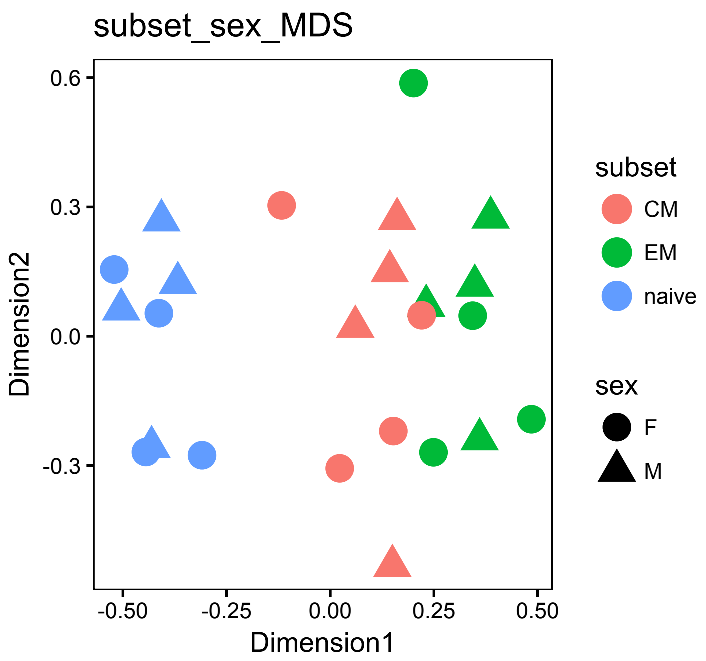

B

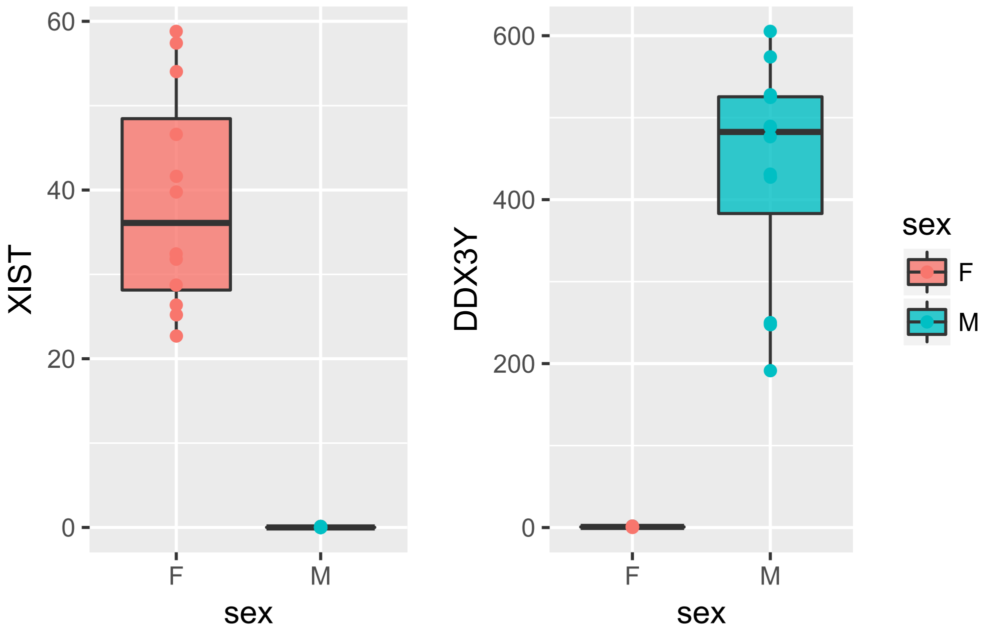

Supplementary Figure 1.
(A) Multi-dimensional Scaling (MDS plot) was used to highlight the transcriptomic variance of the full dataset. Euclidean distance was used and dimension reduction of the whole transcriptome can split the samples based on sorted cell subset. Color represents cell type: blue = naive, red = CM, green = EM. Shape depicts males (triangles) and females (circles). **(B)** Jitter plots highlighting the normalized counts of XIST and DDX3Y in female (red) and male (blue) participants. Gene counts are plotted along the Y-axis and samples phenotype is represented along the X-axis. A Wilcoxon rank sum test was used to assess significance.
