# Supplementary material for: Heightened expression of type I interferon signaling genes in CD4+ T cells from acutely HIV-1–infected women is associated with lower viral loads
Source: Front Immunol. 2025 Jan 20;15:1507530. doi: 10.3389/fimmu.2024.1507530 (PMC11788160; doi:10.3389/fimmu.2024.1507530)
Supplement: Supplementary file 2 [file Image2.pdf]

A

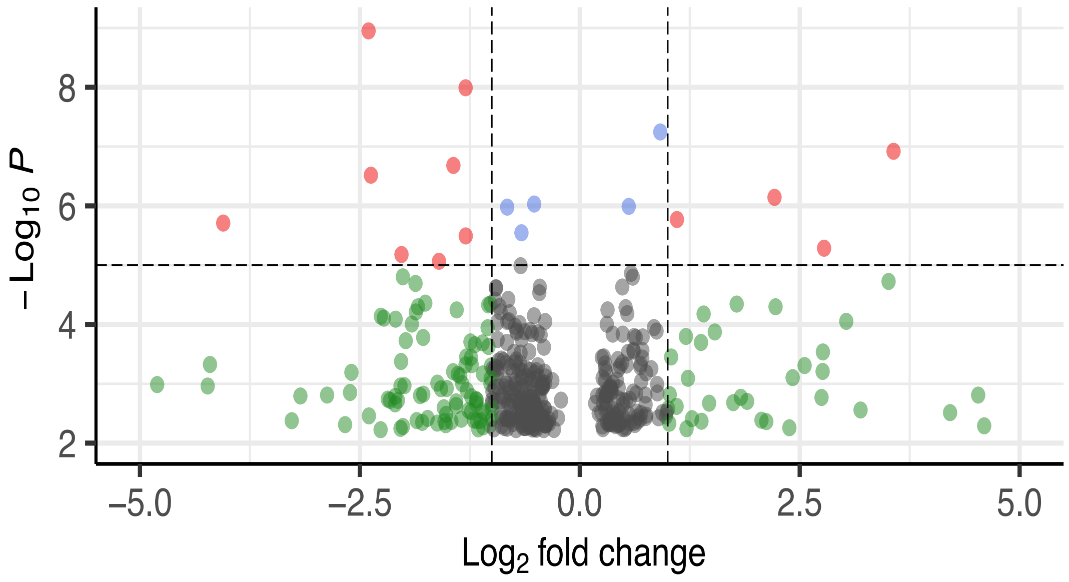

B

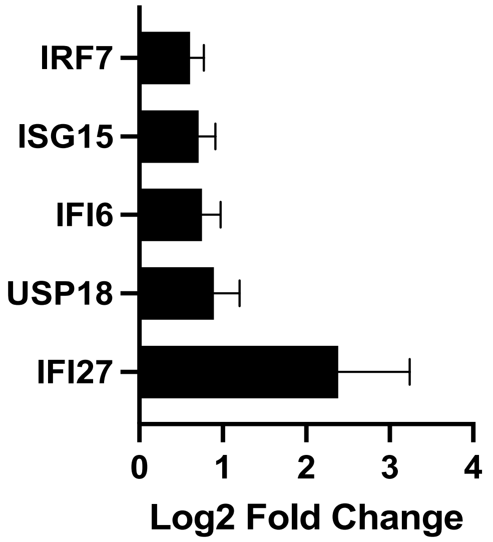

Supplementary Figure 2.
(A) Differential expression of genes in women compared to men (padj<0.20). Genes on X and Y chromosomes are not shown (-Log_{10}P values are beyond the Y axis) **(B)** Type I interferon signaling genes more highly expressed in women.
